# *In silico* identification and characterization of the ion transport specificity for P-type ATPases in the *Mycobacterium tuberculosis* complex

**DOI:** 10.1186/1472-6807-12-25

**Published:** 2012-10-03

**Authors:** Lorena Novoa-Aponte, Andrés León-Torres, Miyer Patiño-Ruiz, Jenifer Cuesta-Bernal, Luz-Mary Salazar, David Landsman, Leonardo Mariño-Ramírez, Carlos-Yesid Soto

**Affiliations:** 1 Chemistry Department, Faculty of Sciences, Universidad Nacional de Colombia, Bogotá, Colombia, Carrera 30 # 45–03, Ciudad Universitaria, Bogotá, Colombia; 2 Computational Biology Branch, NCBI, NLM, NIH, Bethesda, USA; 3 PanAmerican Bioinformatics Institute, Santa Marta, Magdalena, Colombia

**Keywords:** Tuberculosis, *Mycobacterium tuberculosis* complex, P-type ATPases, Ion transport, Conserved motifs

## Abstract

**Background:**

P-type ATPases hydrolyze ATP and release energy that is used in the transport of ions against electrochemical gradients across plasma membranes, making these proteins essential for cell viability. Currently, the distribution and function of these ion transporters in mycobacteria are poorly understood.

**Results:**

In this study, probabilistic profiles were constructed based on hidden Markov models to identify and classify P-type ATPases in the *Mycobacterium tuberculosis* complex (MTBC) according to the type of ion transported across the plasma membrane. Topology, hydrophobicity profiles and conserved motifs were analyzed to correlate amino acid sequences of P-type ATPases and ion transport specificity. Twelve candidate P-type ATPases annotated in the *M. tuberculosis* H37Rv proteome were identified in all members of the MTBC, and probabilistic profiles classified them into one of the following three groups: heavy metal cation transporters, alkaline and alkaline earth metal cation transporters, and the beta subunit of a prokaryotic potassium pump. Interestingly, counterparts of the non-catalytic beta subunits of Hydrogen/Potassium and Sodium/Potassium P-type ATPases were not found.

**Conclusions:**

The high content of heavy metal transporters found in the MTBC suggests that they could play an important role in the ability of *M. tuberculosis* to survive inside macrophages, where tubercle bacilli face high levels of toxic metals. Finally, the results obtained in this work provide a starting point for experimental studies that may elucidate the ion specificity of the MTBC P-type ATPases and their role in mycobacterial infections.

## Background

Tuberculosis (TB) is one of the most important challenges in public health maintenance throughout the world. According to the World Health Organization (WHO), 8.5-9.2 million new TB cases were estimated to have occurred in 2010 [1], and 1.2–1.5 million deaths were caused by species of the *Mycobacterium tuberculosis* complex (MTBC) that includes *M. tuberculosis*, *M. bovis, M. bovis* BCG (vaccine strain), *M. africanum, M. microti, M. canettii,* and *M. pinnipedii,* which produces TB in humans and some animal hosts [2,3]. Part of the infected population will develop active TB, whereas the majority of cases (approximately 90%) progress to a non-infectious disease or latent TB, where mycobacteria survive in a dormant state inside immune cells [4]. Individuals with latent TB may be asymptomatic during prolonged periods of time; however, TB can reactivate when the host immune response diminishes due to malnutrition, steroid use, and HIV co-infection [5].

The emergence of multidrug and extensively drug-resistant tuberculosis strains (MDR-TB and XDR-TB) and the lack of drugs against latent TB have become serious problems for TB control. Therefore, the identification of new therapeutic targets useful in the development of novel drugs and vaccines against latent TB is essential. New anti-TB drugs, such as diarylquinolines (TMC207) and benzothiazines (BTZ043) target essential membrane proteins that affect mycobacterial viability [6]. Thus, antimicrobials designed against proteins of plasma membrane are ideal because they avoid problems related to membrane permeability.

Ion transport in bacteria is carried out by enzymatic systems that belong to either the P-type ATPase, ATP binding cassettes (ABC transporters) and metallic ion/H^+^-antiporter systems [7]. In general, ATPases help maintain the ion gradients responsible for cell volume control and transport of nutrients across the cell membrane [8-11]. ATPases hydrolyze ATP, releasing energy that is used in the transport of ions against electrochemical gradients in plasma membranes. The enzymatic mechanisms of P-type ATPases were initially described in eukaryotic cells [12-14]. These enzymes have five different functional and structural domains: three of these domains are cytoplasmic (A, actuator; N, nucleotide binding and P, phosphorylation), and the other two are embedded in the membrane (T, transport and S, class specific support domain) [15]. P-type ATPases have the following two conformational states: E1, which binds ion substrates on one side of the cell membrane, inducing their auto-phosphorylation and generating a new conformational state; and E2, which has a lower affinity for substrates and therefore releases them to the other side of the cell membrane, promoting ion transport and finally recovery of the E1 state [15,16].

Despite P-type ATPases share the same catalytic mechanism based on conformational changes in their five structural domains, their regulation and substrate affinities are different [15,16]. P-type ATPases are phylogenetically classified into five subfamilies (P_I_-P_V_), and within these subfamilies are 10 different subtypes that are categorized based on the transported substrate [17].

In this study, probabilistic profiles were constructed to compare and classify all P-type ATPases of the MTBC based on their structural features and ion transport. Twelve possible P-type ATPases were detected in the proteome of *M. tuberculosis* H37Rv and from other members of the MTBC. The high number of heavy metal transporters discovered in the MTBC suggests an important role for P-type ATPases in *M. tuberculosis* survival within macrophages.

## Methods

### Construction of hidden Markov models (HMM)

To obtain a representative group of each phylogenetic subfamily, a set of 128 well-characterized P-type ATPases with evidence of existence at the protein level were retrieved from *Uniprot* (Swiss-Prot section) [18]. Each group of sequences was aligned using the *Praline* tool (http://www.ibi.vu.nl/programs/pralinewww/) with the BLOSUM62 matrix and *Phobius* transmembrane structure predictor, which was developed to improve the multiple alignments of membrane protein sequences [19]. The *HMM* package of programs [20], available in the *Mobyle Pasteur* portal (http://mobyle.pasteur.fr/cgi-bin/portal.py#welcome), was used to find these types of pumps in the MTBC proteomes. The *Hmmbuild* tool with default settings and the multiple sequence alignments was used for HMM building. The default parameters of the *Hmmer* tools use an ad hoc position-based sequence weighting algorithm that makes the models appropriate for the identification of distant members of the P-type ATPases family. Consensus sequences were generated with the *Hmmemit* tool.

### Search and classification of MTBC P-type ATPases

To date, the following 10 MTBC genomes have been completely sequenced and assembled (NCBI): *M. africanum* GM041182 (NC_015758), *M. bovis* AF2122/97 (NC_002945), *M. bovis* BCG str. Pasteur 1173P2 (NC_008769), *M. bovis* BCG str. Tokyo 172 (NC_012207), *M. canettii* CIPT 140010059 (NC_015848) and five *M. tuberculosis* strains, H37Rv (NC_000962), H37Ra (NC_009525), F11 (NC_009565), CDC1551 (NC_002755) and KZN1435 (NC_012943); these proteomes were obtained from *Uniprot* (http://www.uniprot.org/). Because strains of *M. microti* and *M. pinnipedii* had not been sequenced, they were not included in this study. HMM and *Hmmsearch* tool were used to find P-type ATPases in the MTBC proteomes.

### Topology prediction

The topology derived from predictions of transmembrane segments (TMS) was made with the following six programs: *TopPred* (http://mobyle.pasteur.fr/cgi-bin/portal.py#forms::toppred), *DAS* (http://www.sbc.su.se/~miklos/DAS/), *TMpred* (http://www.ch.embnet.org/software/TMPRED_form.html), *TMHMM 2.0* (http://www.cbs.dtu.dk/services/TMHMM), *HMMTOP* (http://www.enzim.hu/hmmtop/) and *Phobius* (http://phobius.sbc.su.se/). All programs were used with default settings, except *TopPred*, which allows the user to provide information about the type of organism. *TMDET* and *PPM* servers were used in cases where the predictions based on amino acid sequences did not yield reliable results. These tools must be fed with PDB files to identify TMS based on tertiary protein structure and to generate 3D models with TMS located into hypothetical lipid bilayer planes. Tertiary structure models of CtpH and CtpI were made with the *I-TASSER* tool [21] based on the *threading* strategy and were validated with the *Whatif* package of programs (http://swift.cmbi.ru.nl/servers/html/index.html).

### Hydrophobicity profile construction

The amino acid sequence of the *M. tuberculosis* H37Rv P-type ATPases and consensus sequences generated by *Hmmemit* tool were analyzed by *TMHMM 2.0*. Hydrophobicity profiles of consensus sequences for previously characterized P-type ATPases were used as comparison patterns.

### Conserved motif analysis

Amino acids sequences of the identified proteins were manually analyzed to determine the nine conserved motifs typical of the P-type ATPase family, as previously described by Thever *et al*. [22] and others [16,23].

## Results and discussion

### An unusually high number of cation transporter P-type ATPases are present in the *Mycobacterium tuberculosis* complex

Multiple alignments of 128 reported P-type ATPase protein sequences from a representative group of eukaryotic and prokaryotic cells (obtained from a curated database and confirmed at the protein level) allowed the identification of highly conserved regions within the family and the classification of these members according to ion transport. These alignments were used as the starting point for the construction of HMM profiles that represented groups of ion transporters, which were also used to generate a consensus sequence for each group. The designed HMM were then used to identify P-type ATPases in proteomes of the different MTBC species, whose genome sequences have been reported. The proposed classification for the studied sequences was based on the obtained scores using the HMM and the *Hmmsearch* tool.

Because the P-type ATPases are transmembrane proteins, typical alignments using BLOSUM and PAM matrices were not adequate. In this study, the *Praline* tool was used because it considers the differences in evolutionary tendencies of transmembranal and non-transmembranal regions. *Praline* applies an adequate matrix for each protein region based on the previous prediction of TMS (made with the *Phobius* algorithm in this case). As P-type ATPases contain ion-binding motifs within a transmembrane domain, the strategy that we employed produced more reliable alignments. Twelve hypothetical proteins with a high probability of being P-type ATPase transporters of metallic cations were identified in each of the MTBC proteomes. All proteins identified in the MTBC share at least 98% identity with their orthologs in the scanned proteomes; however, *M. canettii* and *M. africanum* displayed slight differences in non-conserved regions. The pumps identified in the *M. tuberculosis* H37Rv proteome, CtpA, CtpB, CtpC, CtpD, CtpE, CtpF, CtpG, CtpH, CtpI, CtpJ, CtpV and KdpB, were in agreement with the automatic annotation of probable cation transporter P-type ATPases in the *M. tuberculosis* H37Rv genome [24] and were taken as references to facilitate further analysis of results.

### A broad diversity of cations are potentially transported by *Mycobacterium tuberculosis* P-type ATPases

**Figure 1:**
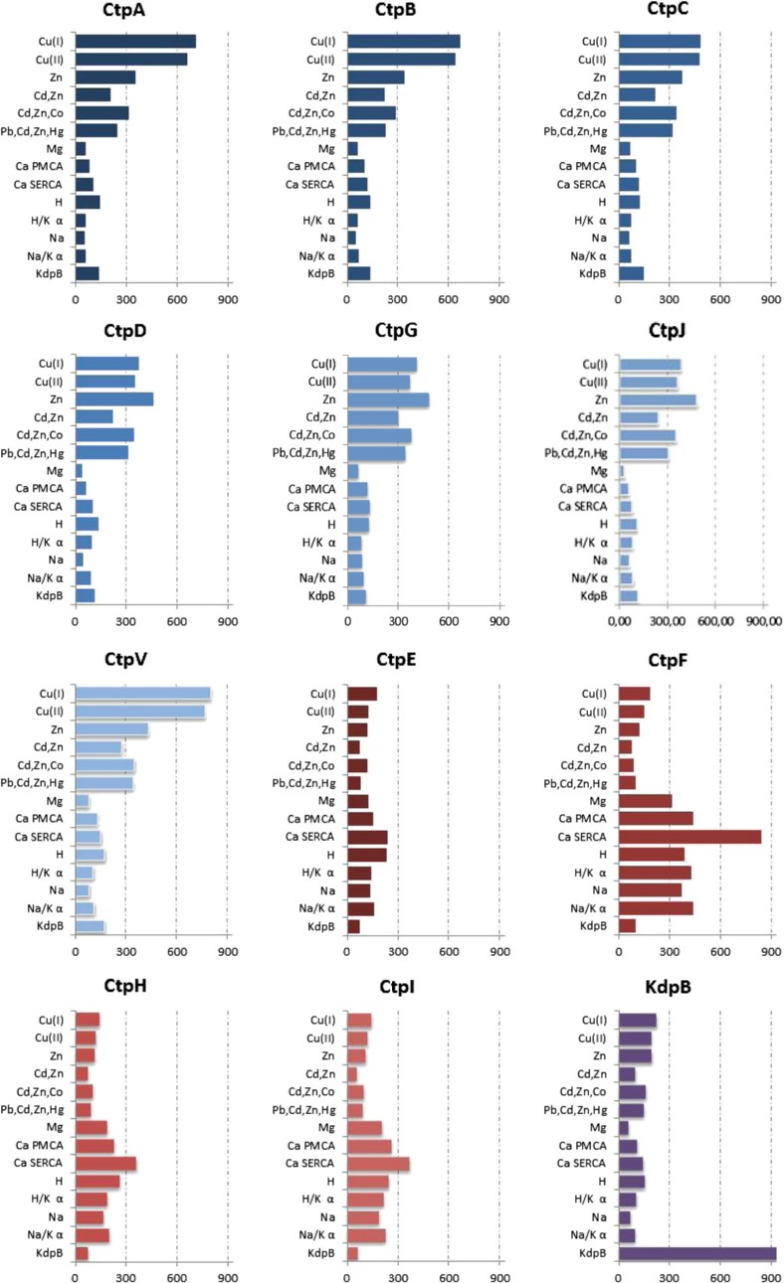
**Classification of P-type ATPases of *M. tuberculosis *H37Rv according to ion specificity.** Amino acid sequences of *M. tuberculosis* H37Rv P-type ATPases were compared with the HMM profiles built from characterized P-type ATPases reported by *Uniprot*. Grouping is based on the scores obtained from *Hmmsearch*. HM group (blue bars) includes the P-type ATPase transporters of heavy metals (CtpA, CtpB, CtpC, CtpD, CtpG, CtpJ, CtpV). The EAM group (red bars) includes transporters of alkaline and alkaline earth metals (CtpE, CtpF, CtpH, CtpI). The KdpB group (purple bars) includes the β subunit of K^+^ P-type ATPase. The scores obtained from the *Hmmsearch* tool are shown in the bottom of each plot.

Figure 1 shows the *Hmmsearch* scores obtained when the 16 HMM were used to find P-type ATPases in the *M. tuberculosis* H37Rv proteome. Those scores show the similarity between the identified sequences in the H37Rv proteome and the grouped sequences used for each HMM construction. The patterns thus obtained allowed classification of the 12 P-type ATPases into the following three groups: transporters of heavy metal (HM) cations, transporters of alkaline and alkaline earth metal (AEM) cations, and a group composed only of the β subunit of the prokaryotic K^+^ transporter, KdpB. The highest similarity with the conventional P-type ATPases used in this study corresponded to KdpB, CtpF and CtpV, whereas CtpE, CtpH and CtpI had the lowest scores.

The HM group is composed of CtpA, CtpB, CtpC, CtpD, CtpG, CtpJ and CtpV, which may transport Cu^2+^, Cu^+^, Co^2+^, Ag^+^, Hg^2+^, Cd^2+^, Pb^2+^ and Zn^2+^. Most of the proteins analyzed in this work correspond to the HM group (60%) suggesting that the active transport of heavy metal cations is relevant for the tubercle bacilli persistence, as it has been hypothesized for other prokaryotes and some unicellular eukaryotes [23]. Interestingly, evidence of toxic concentrations of intracellular heavy metals has been recently described in macrophages infected with *M. tuberculosis*[25].

Alternatively, CtpE, CtpF, CtpH and CtpI belong to the AEM group and may be involved in Na^+^, K^+^, Ca^2+^, H^+^ and Mg^2+^ transport. It is possible that some of these proteins correspond to Na^+^/K^+^ or H^+^/K^+^ ATPase pumps, but they only appeared to contain the α subunits in their tridimensional structure. Their non-catalytic-β-subunit counterpart, which has been correlated with regulatory processes and assembly of P-type ATPases into the cell membrane of eukaryotic cells [10], was not found in any of the MTBC proteomes.

KdpB is very different protein from the other MTBC ATPases. It shares 63% identity with the KdpB of *E. coli* (P03960), which corresponds to the β subunit of a K^+^ transporting multimeric ATPase [22,26]. Despite the observation that KdpB has the characteristic DKTGTLT phosphorylation motif of this type of pump, it does not have a typical ion binding motif [26]. In conclusion, the proposed classification for P-type ATPases of the MTBC provides an initial approximation of their functional characterization. It is noteworthy that the constructed HMM in this work became a useful tool for the identification of P-type ATPases in other biological systems.

### Three different topologies can be adopted by P-type ATPases in *Mycobacterium tuberculosis*

The six different algorithms used in the hydrophobicity analysis showed that all of the *M. tuberculosis* H37Rv P-type ATPases have an α-helix type TMS containing at least 17 amino acid residues, and the agreement of the topology results obtained using these prediction tools increases the confidence in the predicted TMS. Consensus transmembrane regions for each P-type ATPase were obtained if at least four of the algorithms gave similar results. Because three of the tools (*TMHMM 2.0*, *HMMTOP* and *Phobius*) incorporate size and composition restrictions in TMS, the results obtained using these algorithms are significant.

**Figure 2:**
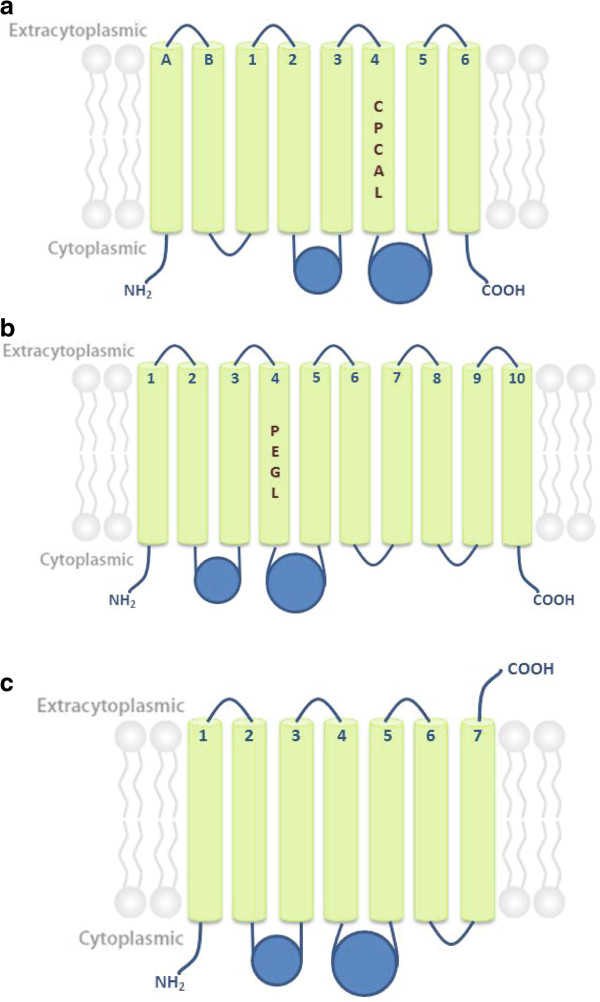
**Predicted topology for P-type ATPases of MTBC.** Type I topology (HM group ATPases) (**a**), type II topology (EAM group ATPases) (**b**), type III topology (KdpB group ATPases) (**c**), Ion binding motifs (number 4) are indicated in the corresponding transmembrane domain.

The strategy used for topology analysis showed the following three different types of topology within the *M. tuberculosis* H37Rv P-type ATPases: type I, which corresponds to HM pumps with eight TMS (A, B and from 1 to 6), type II, which corresponds to AEM pumps with 10 TMS (from 1 to 10), and type III, which corresponds to KdpB with 7 TMS (Figure 2). It was observed that all P-type ATPases have two cytoplasmic loops (small and large) that include the phosphorylation and ATP binding sites. The small cytoplasmic loop is located between TMS2 and TMS3, whereas the largest cytoplasmic loop is situated between TMS4 and TMS5 (Figure 2) [22]. Additionally, as was expected, the N- and C-termini of these proteins are located in the cytosolic side, except in the case of KdpB, in which the N- terminus is located intracellularly and the C- terminus is outside the cytoplasm.

**Figure 3:**
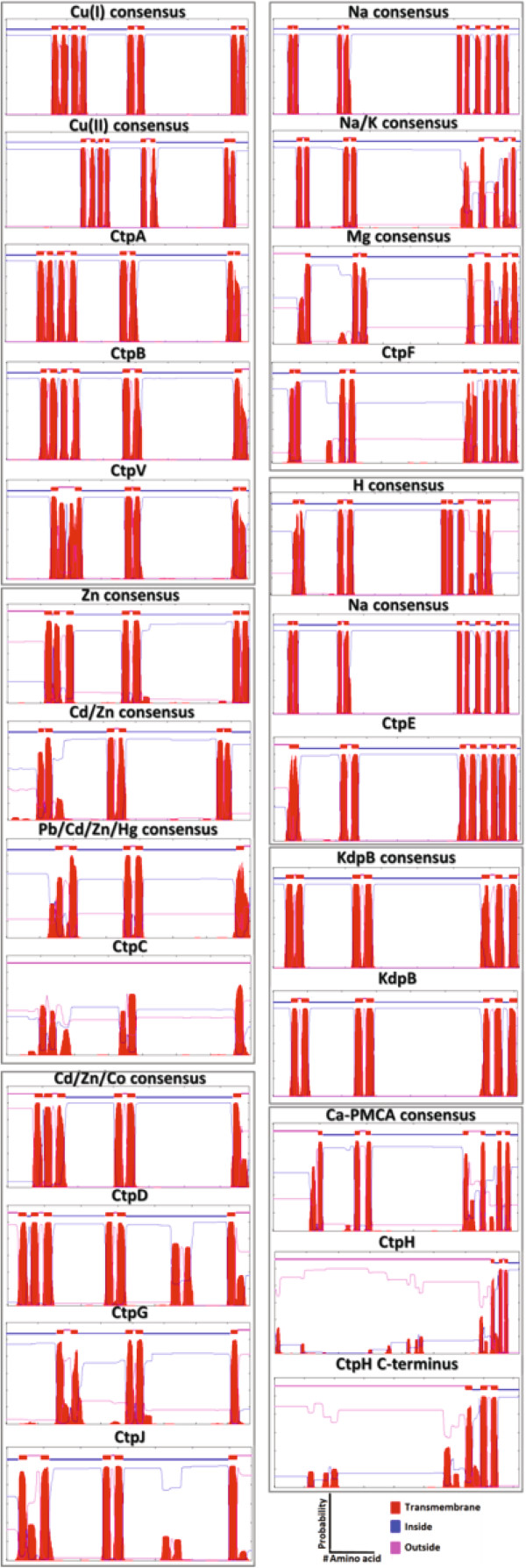
**Hydrophobicity profiles of *M. tuberculosis *H37Rv P-type ATPases.** Hydrophobicity profiles allow grouping of P-type ATPases with seven different ion specificities.

Disagreement in the results was observed in CtpH and CtpI analysis using the different prediction tools. CtpI showed some hydrophobic amino acid sequences that could be considered part of TMS, but they do not fulfill all of the characteristics associated with TMS; meanwhile, CtpH did not show the expected hydrophobicity pattern for P-type ATPases (Figure 3). Therefore, at first it was difficult to determine whether CtpH and CtpI were in fact typical of P-type ATPases. Recent studies have classified CtpH and CtpI proteins of *M. bovis* as FUPA 24 (TC No. 3.A.3.24), i.e., “Functionally uncharacterized P-type ATPase family 24”, and classify them as transporter proteins in the *TCDB-Transporter Classification Database* (http://www.tcdb.org). FUPA 24 proteins are homologous to P-type ATPases but have an unusually large N-terminal segment that makes them twofold longer than typical P-type ATPases [23] with two TMS. In addition, functional motifs of P-type ATPases are located within the C-terminal region of FUPA 24 [23].

**Figure 4:**
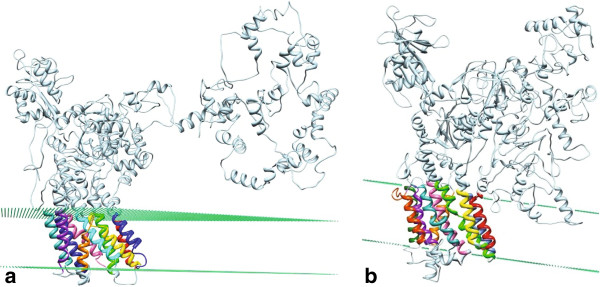
**The CtpH and CtpI mycobacterial P-type ATPases (type II topology) exhibit ten TMS in their C-terminal half (different color helixes).** These tertiary structure models were generated with the *I-TASSER* tool and were modified with the *PPM* server [89] to include the possible location of the lipid bilayer. Dummies (green color) correspond to the location of the carbonyl groups in the bilayer. (**a**) CtpH model generated with the *PPM* server, (**b**) CtpI model built based on the *TMDET* results.

By contrast, topology analysis in this study showed that *M. tuberculosis* H37Rv CtpH and CtpI contain more than the expected TMS (from 3 to 12) for FUPA 24 proteins. To overcome the discrepancy between TCDB and the six topology tools used in this work, the *TMDET* and *PPM* servers were used. These tools allow for the identification of TMS based on the tertiary structure of the protein; thus, not only can the correct size and composition of α-helixes be guaranteed, but also their adequate disposition and organization in the plasma membrane can be determined. Ten TMS were found in CtpH and CtpI using both the *TMDET* and *PPM* servers. These segments are located with high probability in a hypothetical lipid bilayer as shown in Figure 4. This result strongly suggests that CtpH and CtpI have a type II topology, as has been determined for other members of the AEM group.

### Hydrophobicity profiles suggest insights into ion transport specificity

The “topological clustering” based on the hydrophobicity profiles obtained with the *TMHMM 2.0* algorithm can be used as a predictive tool to determine substrate specificity for HM pumps [27]. Here, this strategy was applied to analyze HM, AEM and KdpB groups of *M. tuberculosis* H37Rv P-type ATPases. The hydrophobicity profiles from each *M. tuberculosis* P-type ATPase was compared with the obtained profiles from previously characterized P-type ATPases. As shown in Figure 3, the hydrophobicity profiles of CtpA, CtpB and CtpV were similar to the consensus Cu^+^ P-type ATPases; the CtpC profile was similar to the different Zn^2+^ transporters Zn^2+^, Cd^2+^/Zn^2+^ and Pb^2+^/Cd^2+^/Zn^2+^/Hg^2+^. In addition, CtpD, CtpG and CtpJ had regions similar to the Cd^2+^/Zn^2+^/Co^2+^ transporter. In the case of the AEM group, CtpF possesses TMS that were similar to the hydrophobic profiles obtained for consensus of Na^+^, Mg^2+^ and Na^+^/K^+^ ATPases. Additionally, CtpE was similar to Na^+^ or H^+^ transporters; however, its hydrophobicity profile is closer to that of Na^+^ P-type ATPase. Considering the C-terminus region of CtpH (819 amino acid residues), this pump had a hydrophobicity profile very similar to the Ca^2+^ PMCA consensus. Finally, KdpB had the exact same hydrophobic profile as that which was determined for *E. coli* KdpB.

In general, there are the following two types of hydrophobicity profiles: the first comprises the AEM and the second that corresponds to HM. Interestingly, the relative position of TMS along amino acid sequences is highly conserved between both types of hydrophobicity profiles. The profile for AEM is characterized by a single TMS in the C-terminal region and two in the N-terminal region, whereas profiles for HM P-type ATPases have three highly hydrophobic regions, one of them in the N-terminus, one in the middle of the amino acid sequence, and the third in the C-terminal end. Although the hydrophobicity profile of consensus sequences is similar among enzymes in each group, there are significant differences between profiles that allow their differentiation according to the transported ion.

### P-type ATPases of the *Mycobacterium tuberculosis* complex possess the characteristic motifs involved in cation transport

**Figure 5:**
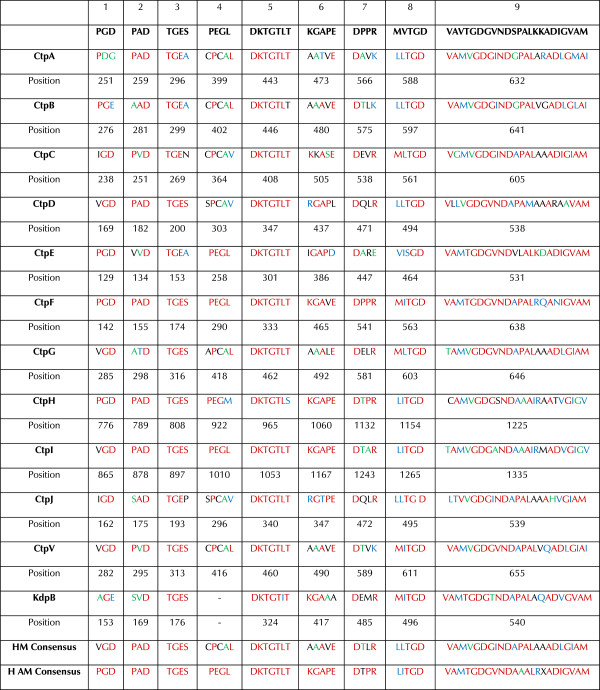
**Conserved motifs for the P-type ATPases identified in *M. tuberculosis *H37Rv.** The conserved motifs were compared with the motifs characteristic of the P-type ATPase superfamily. Identical residues, conserved substitutions, semi-conserved substitutions and unrelated residues are indicated in red, blue, green and black, respectively.

The common catalytic mechanism of P-type ATPases is partially supported by conserved core sequences that are useful in the recognition of these pumps in proteomes [15]. To find the conserved motifs for P-type ATPases along the protein sequences, manual analysis was carried out because the servers commonly used for analysis of conserved motifs do not identify subtle variations within particular motifs of P-type ATPases. Nine conserved motifs typical for P-type ATPases were found in the MTBC pumps (Figure 5). We observed that each motif sequence was identical within orthologs of the MTBC. From N-terminus to the C-terminus, the motifs were as follows: motifs 1 [(PVA)G(DE)] and 2 [P(AS)D], related to conformational changes, together with motif 3 [TGE(SA)], associated with phosphatase activity, were located between TMS 2 and 3 in the actuator domain; the sequence of motif 4, which determines ion specificity, can be [PEG(LM)] or [(CSA)PCA(LV)], which was located at the end of the fourth TMS; the phosphorylation site [DKTGTLT] was found in the P domain; the remaining motifs, motif 6 [(KI)GA(PVA)(EDA)], an ATP binding facilitator, motifs 7 [(DV)(ASPI)(VP)(KAR)] and 8 [(MLV)I(TS)GD], involved in phosphorylation catalysts, and motif 9 [(VTC)AM(TV)GDG(VSAT)ND(AV)(PAL)A(LI)(RKA)(QMAD)A(DNT)(VI)G(VI)(AG)(MV)], the hinge motif, which provides the flexibility necessary to achieve conformational changes during the pumping process, could be found between the fourth and fifth TMS [22]. Motifs 8 and 9 contain amino acid residues [TGDN and GDGXND] responsible for the coordination of Mg^2+^ as a cofactor of the enzymes [15,16,22,23].

It was observed that characteristic motifs of P-type ATPases exhibit slight variations compared with previously reported sequences for eukaryotic organisms [22]. The most conserved motif (motif 5) was almost the same within the 12 P-type ATPases, except CtpH and KdpB, which contained the conservative substitutions [DKTGTL(TS)] and [DKTGT(LI)T], respectively. The sequence of motif 4 [PEGL] has been previously associated with binding of AEM cations [22], and it has been found in CtpE, CtpF, and CtpI. Variation in the fourth position of this motif [PEGM] in CtpH has not been reported previously. CtpA, CtpB, CtpC, CtpD, CtpJ, CtpG and CtpV have the motif 4 sequence [(CSA)PCA(LV)] characteristic of HM transporters. Alternatively, KdpB, which corresponds to a β subunit of a multimeric P-type ATPase, did not have motif 4. This finding can be explained by the observation that this subunit mediates phosphorylation/dephosphorylation and energy transduction during K^+^ transport. In *E. coli*, the coordination function lies mainly in the KdpA subunit [26].

### Cu^+^, Zn^2+^ and Co^2+^ pumping is mediated by P-type ATPases in the *M. tuberculosis* complex

**Figure 6:**
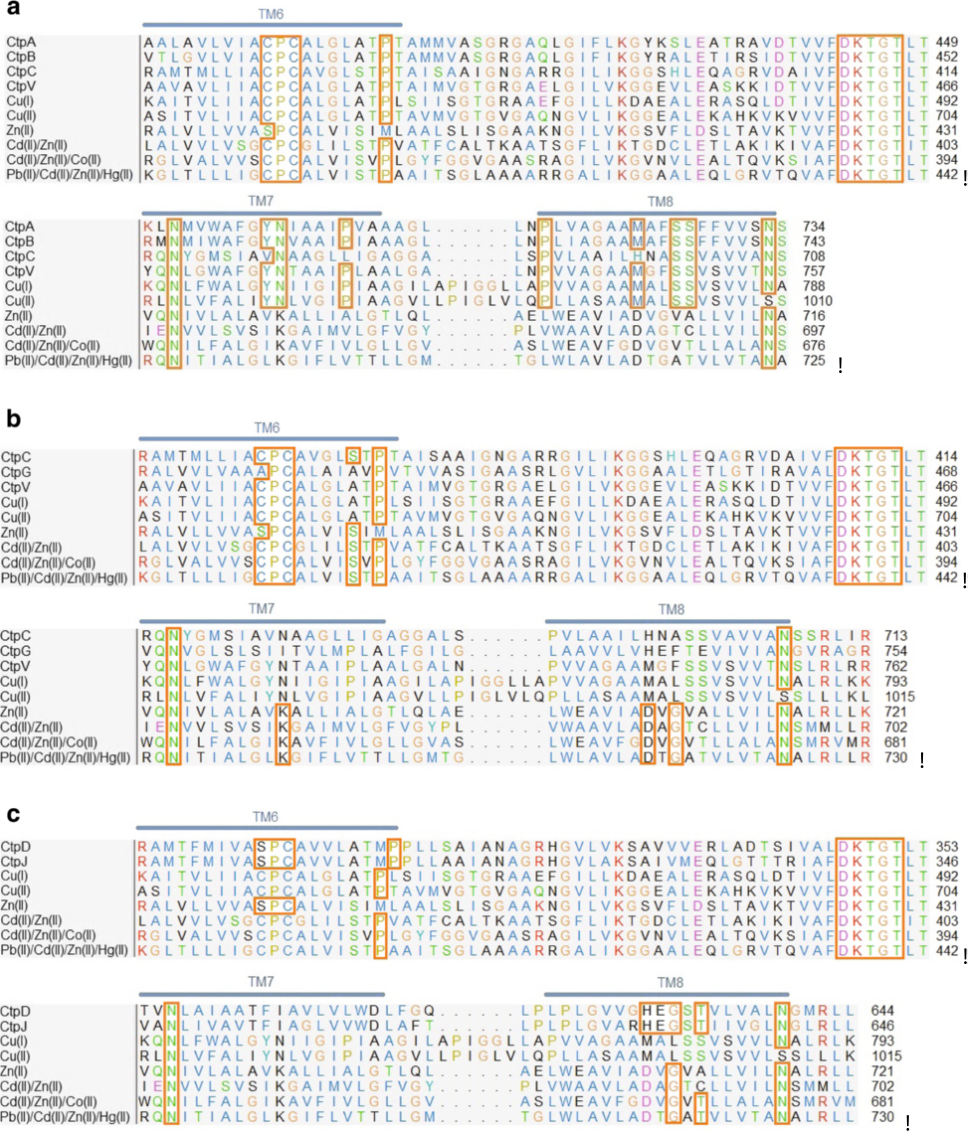
**Alignments of consensus sequences from characterized P**_**IB **_**ATPases, with the HM P-type ATPases from *M. tuberculosis *H37Rv.** (**a**) P_IB-1_ motifs, (**b**) P_IB-2_ motifs and (**c**) P_IB-4_ motifs

The P_IB_ phylogenetic group of P-type ATPases is composed of HM pumps, and according to ion specificity, the P_IB_ group is subdivided into five subgroups (1 to 5). Alignments of consensus sequences from characterized P_IB_ ATPases with the HM P-type ATPases of MTBC were used for locating additional transmembrane motifs of this type of transporter [28-30], as shown in Figure 6. CtpA, CtpB and CtpV have the characteristic motifs of P_IB-1_ group members (Table 1), corroborating their ion transport specificity as predicted by hydrophobicity profiles, and suggesting that Cu^+^ is transported preferentially to Cu^2+^ by these pumps.

**Table 1:** Additional conserved motifs observed in heavy metal transporter P-type ATPases

**Phylogenetic subgroup**	**Cation specificity**	**TMS6**	**TMS7**	**TMS8**	**MTBC pump**
**P**_**IB-1**_	Cu^+^	CPC	YN	MXXSS	CtpA
					CtpB
					CtpV
**P**_**IB-2**_	Zn^2+^	CPC(X)_4_S	K	DG	CtpC *
**P**_**IB-3**_	Cu^2+^	CPH	YN	MXXS	-
**P**_**IB-4**_	Co^2+^	SCP	N	HEGT	CtpD *
					CtpJ *
**P**_**IB-5**_	Undetermined	TPCP	QX_4_GX_3_SX_3_M	PX_5_QEX_2_DX_5_N	-

The additional motifs are located on TMS6, TMS7 and TMS8. *, pumps without a complete set of motifs (see discussion).

This result is expected, because the special reducing conditions inside mycobacteria [27] are similar to the intra-phagosomal environment in macrophages where the bacilli reside. Recently, it was reported that the *M. tuberculosis* H37Rv CtpV is a Cu^+^ exporter P-type ATPase, reinforcing our predictions [31]. CtpD and CtpJ exhibit the [SCP] and [HEGT] motifs of the P_IB-4_ group in the TMS6 and TMS8, respectively. Some reports have indicated that amino acids involved in Co^2+^ transport are still not well understood; for this reason, the absence of residue N in TMS7 cannot rule out the possibility that CtpD and CtpJ are Co^2+^ transporters. CtpC only exhibits the [CPC(X)_4_S] motif in TMS6, which allows its classification as P_IB-2_. In fact, it was recently reported as a Zn^2+^ P-type ATPase in *M. tuberculosis* H37Rv [25]; deficiency in this pump produced zinc accumulation within the mycobacterial cytoplasm, resulting in impaired intracellular growth of tubercle bacilli [25]. Finally, CtpG does not have any of the additional motifs present in TMS6, TMS7 and TMS8; however, it possesses the [WI(YE)(RG)] sequence just before TMS6, between positions 406 and 409, and the [LS] motif located on TMS7, which is associated with Zn^2+^ P-type ATPases [32,33].

All of the results from this work provide predictive evidence for experimental studies to establish the ion specificity of MTBC P-type ATPases and their role in mycobacterial infection. It has been observed that some P-type ATPases of tubercle bacilli are over-expressed under conditions that mycobacteria face during infection [25,34,35]. For example, the expression level of CtpF and CtpC increase when *M. tuberculosis* is exposed to isoxyl, tetrahydrolipstatine and SRI#9190 antimicrobial compounds, indicating that these pumps might contribute to intrinsic resistance of mycobacteria to antimicrobial drugs [36]. Two additional studies indicate that hypoxia induces upregulation of the AEM transporter CtpF [37,38]; this observation is in agreement with those of another study that reported that CtpF is part of the regulon of the DosRS system, a relevant regulator of latency in *M. tuberculosis*[39]. In addition, upregulation of CtpF is observed *in vitro* when *M. tuberculosis* is incubated in the presence of S-nitrogluthatione GSNO, ethanol, H_2_O_2_ and nitric oxide [40]. Moreover, CtpA, CtpC, CtpG, CtpV and CtpF are also induced when *M. tuberculosis* is phagocytized by macrophages [25,34,35].

## Conclusion

Mycobacteria are unicellular organisms that respond to environmental stimuli, and the transport of substances across the plasma membrane could play a fundamental role in their adaptability. Computational analysis shows that each MTBC species has a consistent aggrupation of the 12 P-type ATPases involved in ion transport. In this context, *M. tuberculosis* strains H37Ra and H37Rv share identical sequences for P-type ATPases, facilitating subsequent genetic studies using the attenuated strain H37Ra. The large number of HM P-type ATPases expressed by the MTBC strongly suggests that they could be essential for the bacteria to counteract the increased level of HM accumulated by macrophages after infection with tubercle bacilli. Thus, compensatory ion transport strategies could be used by mycobacteria to survive in host cells.

The different bioinformatics approaches used in this work to analyze the P-type ATPases identified in the MTBC are in agreement with the initial classification from the HMM search. The results obtained show that *M. tuberculosis* has the following three groups of P-type ATPases: HM transporters (CtpA, CtpB, CtpC, CtpD, CtpG, CtpJ and CtpV), AEM transporters (CtpE, CtpF, CtpH, and CtpI) and the KdpB protein, which corresponds to the β subunit of a multimeric K^+^ ATPase transporter exclusive to prokaryotes. Hydrophobicity analysis identified α-helix type TMS grouped into the following three topological types: type I (HM group), type II (AEM group) and type III (KdpB group). Interestingly, we report a possible mis-annotation for CtpH and CtpI in the TCDB Database, where they are classified as FUPA 24 type with two TMS, unlike the ten TMS identified for these unusually large transporters in this work. Finally, a counterpart of non-catalytic β subunits of Na^+^/K^+^ or H^+^/K^+^ ATPases does not exist within the MTBC proteomes.

## Competing interest

There are no conflicts of interest to declare.

## Authors' contributions

LN-A, AL-T, MP-R, JC-B, L-M S, DL, LM-R and CY-S wrote the manuscript, validated the tools and carried out the data analysis and interpretation. LN-A, AL-T, MP-R and JC-B contributed to the methodological design, supervised its development and critically revised the manuscript's content. All authors read and approved the final version of the manuscript.
